# Supplementary material for: Impact of depression on personal hygiene practices- A cross-sectional study among university students in Bangladesh
Source: PLoS One. 2025 Nov 18;20(11):e0323521. doi: 10.1371/journal.pone.0323521 (PMC12626304; doi:10.1371/journal.pone.0323521)
Supplement: S1 Table — (DOCX) [file pone.0323521.s001.docx]

**S1 Table:** Comparison of Binary Logistic Regression of Personal Hygiene Coefficients to Assess Proportional Odds Assumption

| Variable | Poor vs. Moderate/Good Personal Hygiene | Poor/Moderate vs. Good Personal Hygiene |
| --- | --- | --- |
|  | Coefficients with p-values | Coefficients with p-values |
| At-risk depression | -1.06 (p < 0.001) | -1.01 (p < 0.001) |
| Male gender | -1.12 (p < 0.001) | -1.15 (p < 0.001) |
| University type (private) | -0.05 (p = 0.772) | 0.02 (p = 0.884) |
| Second year (vs. 1st) | -0.45 (p = 0.021) | -0.40 (p = 0.025) |
| Residence (private) | +0.68 (p < 0.001) | +0.70 (p < 0.001) |

We categorized the personal hygiene variable into binary and then ran separate binary logistic regression to check if we could use ordered logistic regression analysis. The nearly similar directions, coefficients and p-values for depression, gender, year, and residence across the two models showed that proportional odds assumption is met for us to use ordinal logistics regression analysis.

**Limitation Statement for the Violation of Proportional Odd Assumption for “University Type” Variable:**

We included the “university type” variable as a predictor in the overall ordered logistic regression analysis in spite of the slightly changed directional effect (from -0.05 to 0.02). We were concerned about slight violation of the proportional odd assumption for this variable. However, in the ordered logistic regression analysis, this variable was not significantly associated (p-value of 0.955) with the outcome. Therefore, we acknowledge the slight violation of the proportional odd assumption for the “university type” variable, it is not likely to impact the overall results.
