# Supplementary material for: Impact of depression on personal hygiene practices- A cross-sectional study among university students in Bangladesh
Source: PLoS One. 2025 Nov 18;20(11):e0323521. doi: 10.1371/journal.pone.0323521 (PMC12626304; doi:10.1371/journal.pone.0323521)
Supplement: Attachment — Submitted filename: Response to reviewer1.docx [file pone.0323521.s004.docx]

**Author Response to Reviewer**

**Manuscript ID:** PONE-D-25-16896

**Manuscript Title:** Impact of depression on personal hygiene practices- A cross-sectional study among university students in Bangladesh

**Reviewer Comment:** **Unspecified sampling method**: The method used (e.g., convenience, snowball, stratified) is not clearly stated.

**Authors Response:** “We used a convenience sampling method to recruit participants for the study.”

**Reviewer Comment:** **Generalized claim of diversity**: The phrase *"to ensure sample diversity"* lacks evidence—what dimensions of diversity were targeted (e.g., university type, gender, department)?

**Authors Response:** “A structured questionnaire was distributed via a Google Form link to ensure accessibility for participants across various locations with the study nature, purpose and eligibility inclusion criteria for participation. The inclusion criteria required for the participants to be 1) a resident in Dhaka, 2) studying either public or private university, 3) studying in first/second/third/fourth year, 4) studying health or non-health background subject majors, 5) have access to computer or mobile with internet connection, 6) able to understand English and 7) written consent for participation ... Participation was voluntary, students who were interested accessed and completed the questionnaire. We shared invitations for participation in this study across both public and private university networks, student groups, and social media platforms.”

**Reviewer Comment:** **Non-standard date formatting**: Dates like "08/09/24" should follow academic standards (e.g., *September 8, 2024*).

**Authors Response:** “Recruitment of participants for data collection started on September 8, 2024, and ended on December 31, 2024.”

**Reviewer Comment**: **Missing response rate**: No mention of how many students were invited or the response rate, which affects generalizability.

**Authors Response:** Thank you for your valuable comment. We have corrected the information.

“The survey link was shared online on different platforms. Initially, 2,030 participants provided written informed consent online. After applying exclusion criteria for missing values and inconsistencies, 1913 respondents completed the entire survey, generating a response rate of 94.23%.”

**Reviewer Comment**: **Vague terminology**: *"individual’s subject major"* should be replaced with *"students’ field of study"*.

**Authors Response:** The socio-demographic section contained data on age, gender, type of university, students’ field of study and their level of study, place of residence, parental education, and family income in Bangladeshi currency (BDT).

**Reviewer Comment:** **Income categorization**: The quartile method for income is mentioned but not clearly explained—what ranges were used?

**Authors Response:** “We used the quartile method based on participant’s self-reported monthly family income and divided the continuous income variable into four quartiles, where each quartile represents 25% of the sample. The cut-off value of each quartile was determined by Stata based on the distribution of family income variable in our dataset.”

**Reviewer Comment:** **Missing translation details**: It is unclear whether the CES-D scale was used in English or translated into Bengali, and if so, whether it was culturally validated.

**Authors Response:** We didn’t translate the CES-D scale in Bengali as one of our inclusion criteria for the participant was to be able to understand English. As the CES_D scale was not translated, further validation wasn’t required.

**Reviewer Comment:** **Mixed response scales**: Using both 3-point and 4-point Likert scales can confuse respondents and complicate scoring and analysis.

**Authors Response:** We used a combination of 3-point and 4-point Likert response scales in our questionnaire based on the nature of the items and expert input.

After developing the questionnaire, a pilot survey was conducted with 44 students to check whether they could easily understand the questions and response options. 93.18% of students didn’t find any difficulties in understanding the question and 86.36% of students had no issues with the options of the questions.

**Reviewer Comment:** **Lack of clear construct validation**: Although Cronbach’s alpha is provided, there’s no detail on *when* exploratory factor analysis (EFA) was conducted—before or after analysis?

**Authors Response:** To assess construct validity, we conducted Exploratory Factor Analysis (EFA) using principal factor extraction and varimax rotation before conducting the regression analysis.

**Reviewer Comment:** **Limited detail on development process**: While expert review and a pilot test were mentioned, there's no detailed breakdown of how items were selected, modified, or validated.

**Authors Response:** During questionnaire development, we initially created 21 items based on literature review. After consultations with faculty members from nutrition, pharmacy, and public health, we removed three items (questions on deodorant apply, wash own clothes, and use of tissue for nose picking) that were deemed redundant or less relevant, resulting in the final 18-item version. A pilot test among 44 students confirmed the clarity and appropriateness of items, and wording was refined based on student feedback.

**Reviewer Comment:** **Scoring scheme complexity**: The scoring rules are somewhat convoluted and may introduce bias or inconsistency.

**Authors Response:** We acknowledge the complexity in the use of both 3-point and 4-point Likert scales in the questionnaire. To maintain consistency and reduce potential bias in scoring, responses from both 3-point and 4-point Likert scales were recoded into a common scale of 0, 1, and 2.

Items on a three-point scale were scored as (i) "Always" = 2, "Sometimes" = 1, "Never" = 0; (ii) "Daily" = 2, "Weekly" = 1, "Monthly" = 0.

For the four-point scale, responses were coded as "Once a week/Once in 15 days" = 2, "Once a month" = 1, and "Once more than one month duration" = 0.

**Reviewer Comment:** **Repetition**: Cronbach’s alpha is mentioned multiple times unnecessarily.

**Authors Response:** Corrected in the result section.

**Reviewer Comment:** **Minor terminology error**: *"logistics regression"* should be *"logistic regression"*.

**Authors Response:** Corrected

**Reviewer Comment:** **Assumption checks missing:** No mention of whether model assumptions (e.g., proportional odds for ordered logistic regression) were tested.

**Authors Response:** Thank you for the comment. We have done the assumption test for ordered logistic regression. We have added the table in the supplementary documents (S1 Table).

**Reviewer Comment:** **Weak sequencing**: Statistical methods could be better organized—from descriptive stats to inferential analysis.

**Authors Response:** We have organized the statistical methods from descriptive to inferential analysis in the revised version. Thank you.

**Reviewer Comment:** **Grammatical issues**: Several grammatical and phrasing errors reduce clarity (e.g., plural agreement, missing articles).

**Authors Response**: Corrected

**Reviewer Comment:** **Casual or unclear expressions**: Some sentences (e.g., “we got a value of...”) lack academic tone.

**Authors Response:** Corrected

**Reviewer Comment:** **Lack of cohesion**: Transitions between sections are abrupt and need better integration.

**Authors Response:** We have revised the manuscript and maintain the transitions for better integration.

**Reviewer Comment:** **Lack of Clear Structure:**

The discussion lacks a well-defined structure. Topics such as depression, personal hygiene, tool validation, and limitations are interwoven without clear thematic separation, making it harder to follow the argument logically.

**Authors Response:** Thank you very much, we have corrected the structure of the discussion section by following to follow the argument logically:

1. Describe the prevalence and associated factors of depression and personal hygiene
2. Then we describe the major findings of our study which is the association of depression with personal hygiene
3. And, then we discuss about the validation of our newly developed personal hygiene scale.

After that we discuss about the strengths and limitations of the study.

**Reviewer Comment:** **Redundancy and Awkward Phrasing:**
Some sentences are repetitive or awkwardly constructed. For example:

*"We also found a high prevalence of depression risk among male and female students but comparing with male, female students had a higher-level depression risk."*
This could be rewritten more clearly as:
*"Depression risk was high among all students, but higher among females than males."*

**Authors Response:** Thank you very much for the comments. We have corrected and paraphased the sentences that are repetitive and awkwardly constructed.

**Reviewer Comment:** **Superficial Analysis of Relationships:**

While statistical associations are mentioned, the discussion lacks in-depth theoretical or psychological explanations for the observed relationships (e.g., how and why depression affects hygiene behaviors). Including relevant behavioral or mental health theories would strengthen the interpretation.

**Authors Response:** Thank you for the comments.

We tried to write the discussion providing some theoritical explanation that may contribute to estabilishing the relationship between mental health and personal hygiene.

**Reviewer Comment:** **Insufficient Exploration of Contradictory Findings:**

The study acknowledges discrepancies with previous research (e.g., gender differences in depression), but does not delve deeply into possible reasons for these inconsistencies, such as cultural context, sample differences, or measurement tools.

**Authors Response:** Thank you very much for the comments. We have added some line describing the reasons behind the differences between the present studies with previous studies.

**Reviewer Comment:** **No Conceptual Framework Provided:**
The study would benefit from presenting a conceptual model that visually or descriptively outlines the relationships between depression, hygiene practices, and demographic/socioeconomic factors.

**Authors Response**: We have rewrite the discussion to provide a clear understanding and logical arguments and try to present a conceptual model to define the relationships between personal hygiene, depression and other socio-demographic characteristics.

**Reviewer Comment:** **Lack of Concrete Recommendations**
Although implications are hinted at, the discussion does not clearly provide practical recommendations for universities, policymakers, or future researchers based on the study’s findings.

**Authors Response:** Thank you very much for the comments. We have added some recommendations for the universities, policy makers and future researchers in the conclusion part.

**Reviewer Comment:** **Overstatement of Causality**:

“Reduced incidence of depression and higher hygiene standards”
The cross-sectional design does not allow causal claims. Phrases like “showed a reduced incidence of depression” or “leads to improved health outcomes” should be softened (e.g., “were associated with…”).

**Authors Response:** Thank you very much for the concern. We have correct the statement and remove the overstatement of causality from the conclusion part.

**Reviewer Comment:** **Overly Idealistic Recommendations**:
While workshops and screenings are valuable, the text assumes feasibility and efficacy without considering potential barriers (e.g., resource constraints, stigma, institutional policy gaps). These should be acknowledged.

**Authors Response:** Thanks to the reviewer. We have acknowledged the different barriers related to hygiene and depression at social and instutional level.

“Along with the educational programs, barriers related to social and institutional also need to be focused to find out the gaps in policy making by government.”

**Reviewer Comment:** **Generalization to Broader Populations**:
Statements like “we can enhance the overall well-being of students” should be more cautious, given that the data are limited to a narrow student demographic in Dhaka.

**Authors Response:** Thank you. We have removed the statements.

**Reviewer Comment:** **Lack of Linkage Between Findings and Interventions**:
The proposed interventions (e.g., peer support) are not clearly tied back to specific findings from this study. It would strengthen the conclusion to align each recommendation with a corresponding empirical result.

**Authors Response:** We have rewrite and added some lines to provide strengths in conclusion of the recent study.
