# Supplementary material for: Impact of depression on personal hygiene practices- A cross-sectional study among university students in Bangladesh
Source: PLoS One. 2025 Nov 18;20(11):e0323521. doi: 10.1371/journal.pone.0323521 (PMC12626304; doi:10.1371/journal.pone.0323521)
Supplement: Attachment — Submitted filename: Response to Reviewers.docx [file pone.0323521.s005.docx]

**Author’s Response to Reviewer’s Comments**

**Manuscript ID:** PONE-D-25-16896R1

**Manuscript Title:** Impact of depression on personal hygiene practices- A cross-sectional study among university students in Bangladesh

**Reviewer’s Comment:** Ensure that all claims, especially those related to prevalence rates and contributing factors to depression, are up-to-date and supported by the most recent literature.

**Author’s Response:** Thank you very much. We have used recent literature (2020 to 2025) for reporting prevalence and other contributing factors related to depression and personal hygiene as much as possible.

**Reviewer’s Comment: Literature Review**: A more comprehensive literature review is needed. The introduction references several studies, but does not adequately synthesize findings or highlight gaps in the research. Discussing existing theories or models that relate to the relationship between hygiene and mental health would provide a solid theoretical framework.

**Author’s Response:** Thank you very much. We have highlighted the gaps found in different literatures and mentioned those in the introduction part to strengthen the study’s aim.

**Reviewer’s Comment: Sampling Bias:** The use of convenience sampling can lead to bias. The article should clarify any efforts made to recruit a representative sample, and discuss the implications this has for generalizability of findings. Overall, the methodology presented is thoughtfully developed with appropriate measures and analyses. Addressing the mentioned areas of improvement, particularly the discussion of sampling limitations, detailed statistical methods, and qualitative feedback from pilot testing, would enhance the clarity and robustness of the methodology section.

This will provide readers a clear understanding of the study's validity and reliability and potentially improve the study's impact in the field.

**Author’s Response:** We agree with the reviewer that the use of convenience sampling may introduce sampling bias and limit the generalizability of our findings. In our study, we tried to reduce this limitation by sharing the survey link in both public and private university networks, with the male and female student groups, and among students from both health and non-health backgrounds. Using this approach, we were able to identify variation across university types, gender, and academic disciplines. As the participants are self-selected into the study, the sample might not completely represent all university students in Dhaka. We have now added a clear statement in the methodology and discussion sections to acknowledge this limitation and to explain that our findings should be interpreted with caution when generalizing to the wider student population.

**Reviewer’s Comment:** The discussion around cultural influences on hygiene practices and mental health in Bangladesh is limited. An exploration of local cultural factors that influence both personal hygiene and mental health would enrich the discussion.

**Author’s Response:** Some lines about cultural influences have been added although there are limited literatures that evaluated the effect of cultural influences on personal hygiene or mental health. Thank you.

**Reviewer’s Comment:** The article does not sufficiently address potential confounding variables other than gender and accommodation type. Socioeconomic factors and lifestyle variables may confound results and should be critically analyzed.

**Author’s Response:** Thank you for the comment. We have adjusted for potential confounders (age, gender, university type, subject major, year of study, residence type, parental education, and family income quartile) in the regression analysis but wasn’t clearly mentioned in the write-up**.** Now, in the statistical analysis and result section of the revised manuscript, we have revised the write-up and clarified the confounders included in our regression analyses.

**Reviewer’s Comment: Discussion of Findings:** While the results highlight statistical significance, the discussion should focus more on the practical implications of these findings. What does this mean for university policy or mental health interventions?

**Author’s Response:** Thank you for the comments. We have added some practical implications line in the discussion section.

**Reviewer’s Comment: Word Choice and Clarity:** The use of the phrase "personal hygiene is crucial for maintaining overall health" should be evaluated; it can be too broad. A more precise statement focusing on mental health would be beneficial.

**Author’s Response:** We have read the overall manuscript again, and rewrite and changes the word where was necessary to clarify the sentences for reader.

**Reviewer’s Comment:** Minor grammatical issues should be rectified, such as ensuring consistency in the use of singular/plural forms throughout the text.

**Author’s Response:** We have read the manuscript and correct the grammatical mistakes and typos.

**Reviewer’s Comment:** The references should be uniformly formatted according to the journal's style. They appear to be inconsistently presented throughout the document.

**Author’s Response:** Thank you very much. We have rechecked every reference for uniformity and maintained the journal guideline.

**Reviewer’s Comment:** Abbreviations like PHPQ and CES-D should be defined at first use in the abstract or introduction, and then used consistently thereafter.

**Author’s Response:** Thank you for the comment. We have defined the abbreviation at first use and used them consistently thereafter.

**Reviewer’s Comment:** A dedicated limitations section could strengthen the paper. Discussing limitations related to the cross-sectional design, self-reported measures, and any potential biases would enhance transparency.

**Author’s Response:** We have included a dedicated limitation section.

**Reviewer’s Comment:** The conclusion repeats the findings from the results rather than synthesizing them into broader implications. It should provide a more rounded perspective on how the findings could impact policies or practices.

**Author’s Response:** We have organized and added some lines to provide broader implications of our study findings in the conclusion part.
